# Smaller gray matter volume of hippocampus/parahippocampus in elderly people with subthreshold depression: a cross-sectional study

**DOI:** 10.1186/s12888-016-0928-0

**Published:** 2016-07-07

**Authors:** Huixia Zhou, Rui Li, Zhenling Ma, Sonja Rossi, Xinyi Zhu, Juan Li

**Affiliations:** Center on Aging Psychology, Key Laboratory of Mental Health, Institute of Psychology, Chinese Academy of Sciences, 16 Lincui Road, Chaoyang District, Beijing, 100101 China; Magnetic Resonance Imaging Research Center, Institute of Psychology, Chinese Academy of Sciences, 16 Lincui Road, Chaoyang District, Beijing, 100101 China; School of Nursing, Peking Union Medical College, Beijing, China; Clinic for Hearing-, Speech- and Voice Disorders, Medical University of Innsbruck, Innsbruck, Austria

**Keywords:** Subthreshold depression, Older adults, Hippocampus/parahippocampus, Gray matter volume

## Abstract

**Background:**

Hippocampal/parahippocampal structural changes accompany major depressive disorders in the elderly, but whether subthreshold depression (StD) at an advanced age is also accompanied by similar changes in hippocampal/parahippocampal volumes is still unknown. By using voxel-based morphometry (VBM) analysis of the gray matter, we explored whether there are structural alterations of the hippocampus/parahippocampus and the correlations between its volume and participants’ self-reported depressive symptoms.

**Methods:**

Participants were 19 community-dwelling older adults with StD assessed by the Center for Epidemiologic Studies Depression scale (CES-D) scores. We collected magnetic resonance images of their brain compared to images of 17 healthy aged-matched adults. We used VBM to analyze differences in gray matter volume (GMV) of the hippocampus/parahippocampus between the two groups. Moreover, we examined the correlation between the GMV of the hippocampus/parahippocampus and participants’ self-reported depressive symptoms.

**Results:**

VBM revealed that elderly individuals with StD had substantially reduced volumes of the right parahippocampus compared to healthy controls. Furthermore, the volumes of the hippocampus/parahippocampus were significantly associated with participants’ self-reported depressive symptoms in StD.

**Conclusions:**

Gray matter volume alterations in the hippocampus/parahippocampus are correlated with subthreshold depression suggesting that early structural changes in the hippocampus/parahippocampus can constitute a risk indicator of depression.

**Electronic supplementary material:**

The online version of this article (doi:10.1186/s12888-016-0928-0) contains supplementary material, which is available to authorized users.

## Background

Depression is one of the most prevalent mental disorders in elderly people over 60 years of age, affecting 10 to 15 % of the aging population [1, 2]. Depression in the elderly is characterized by various etiological factors that remain poorly understood [3, 4]. Successes in delineating the neurobiology of depression have closely paralleled progress in neuroimaging and have provided evidence that depression is associated with underlying brain anatomic and functional abnormalities in cortical-limbic-subcortical circuit [5, 6].

The hippocampus (HC) and parahippocampus (PHC) that located in the medial temporal lobe (MTL) are essential parts of the cortical-limbic-subcortical circuit, which consists of the frontal cortex, cingulate cortex, amygdala, hippocampus, and other subcortical structures [7]. This brain network usually regulates emotional behavior thus playing a central role in the pathophysiology of major depressive disorders (MDD) [7–9]. Neuroimaging studies emphasized the importance of the hippocampus in the pathophysiology of MDD in older adults [5, 10, 11]. Particularly, hippocampal volume anomalies in older patients suffering from MDD have been repeatedly reported [12–16]. It has also been proposed that volumetric abnormalities in the parahippocampus lead to impairments of the cortical-limbic-subcortical circuit and thus structural changes of the parahippocampus have been found to be connected to the etiology of depression at an advanced age [5]. Reduced hippocampal-dorsolateral prefrontal cortex (DLPFC) connectivity was also found in older adults with depression [17]. In addition, elderly people suffering from subthreshold depression (StD), a disorder characterized by above-average self-reports of depressive symptoms however not fulfilling the diagnostic criteria for MDD [18], demonstrated decreased hippocampal/parahippocampal connectivity to the superior and medial frontal gyri compared to normal controls [19]. These results suggest that top-down control of the prefrontal cortex over limbic regions is attenuated in older adults with depression or StD, which may cause depression [7, 9, 20].

Although many studies found structural abnormalities of the hippocampus/parahippocampus in older adults with MDD, only two studies investigated the role of structural alterations in elderly persons suffering from StD. These studies, however, failed to find hippocampal/parahippocampal volume reductions [21, 22]. Possible explanations for these negative findings might be found in differences in study samples, employed measurements for assessing depression, the imaging protocols, and also the usage of different analytic strategies. As a consequence, it remains unclear whether structural abnormalities in the hippocampus/parahippocampus emerge in elderly people with StD. More intense research on this topic is thus necessary.

StD is more prevalent than MDD in the elderly [18], which has been shown to seriously affect patients’ quality of life and daily functioning and also to increase health service burden [23, 24]. Individuals with StD can be situated in the midway of a continuum ranging from a normal affect to a clinically relevant depression. In the case of prodromal depression, adults with StD would progress to clinical depression [25]. Thus, studying and understanding the neuronal bases of these mechanisms is essential to be able to predict a progression toward a clinically relevant depressive episode. Identification of MRI (Magnetic Resonance Imaging) biomarkers of individuals with StD will thus provide important insights affecting an early diagnosis of individuals at-risk and possibly allowing prevention of depression onset.

In summary, the goal of the current study is to explore whether structural alterations of the hippocampus/parahippocampus and correlations between its volume and participants’ self-reported depressive symptoms could be identified in elderly people suffering from StD. For this purpose, voxel-based morphometry (VBM) will be used for a region-of-interest analysis to detect gray matter volume alterations in elderly StD subjects compared to healthy age-matched controls.

Based on the findings of previous structural imaging studies with psychiatric samples, we hypothesize that older adults with StD would disclose hippocampal/parahippocampal volume changes and that these abnormalities might relate to self-reported depressive symptoms.

## Methods

### Participants

In order to recruit old adults with StD, we screened 1100 subjects in local communities of Beijing, China. Although 20 % of them fulfilled the criteria we defined for StD, the majority of them cannot participate the experiment because of poor body condition, such as cardio-cerebrovascular disease, disabled in mobility, presence of metal in the body and so on. Therefore, only 19 elderly subjects with StD were selected from them. 17 healthy control older adults were also recruited from the same sources as StD participants. All participants received a psychological health lecture from investigators followed by a survey using the Center for Epidemiologic Studies Depression scale (CES-D) [26]. The surveys were assessed by a licensed psychiatrist. According to previous studies [27–30], all elderly, who scored 8 or more on CES-D and did not meet the DSM-IV (Diagnostic and Statistical Manual of Mental Disorders - IV) diagnostic criteria for major depression were selected as StD participants. In comparison, all HC participants did not have any history of depression and scored five or less on CES-D. A Mini Mental State Examination (MMSE) cutoff of 24 or more [28, 29, 31] was used to exclude any potential cognitive impairment in all participants in both groups. None of the participants in either group had a history of central nervous system disease, mental illness, or serious head trauma.

### MR image acquisition

Brain MR Images were obtained using a 3-T Siemens scanner at the Imaging Center for Brain Research in Beijing Normal University. A high-resolution, three-dimensional sagittal T1-weighted structural image was acquired for each subject with the following parameters: 176 slices; resolution 256 × 256; voxel size 1 × 1 × 1 mm^3^; TR 1900 ms; TE 2.2 ms; and flip angle 9°.

### Image analyses

All the three-dimensional structural MR images were normalized and segmented into gray matter (GM), white matter, and cerebrospinal fluid (CSF) by using the NewSegment and DARTEL modules included in the Statistical Parametric Mapping program (SPM8, Wellcome Trust Center for Neuroimaging, London, UK, http://www.fil.ion.ucl.ac.uk/spm), the Data Processing Assistant for Resting-State fMRI (DPARSF, advanced edition, http://rfmri.org/DPARSF) and Resting-State fMRI Data Analysis Toolkit (REST, http://restfmri.net/forum/REST_V1.8) run on MATLAB R2012b on a Windows desktop computer. As an extension of the “unified segmentation algorithm”, the new Segmentation model includes additional tissue probability maps to better model CSF and other non-brain voxels, thus resulting in a more precise segmentation.

Following segmentation, the GM segments of all 36 participants in both groups were processed to create study-specific GM population templates using DARTEL algorithm. After an initial affine registration of the GM DARTEL template to the tissue probability map in the Montreal Neurological Institute (MNI) space (http://www.mni.mcgill.ca/), non-linear warping of GM images was performed to the DARTEL GM template in the MNI space and resampled to an isotropic resolution of 1.5-mm^3^. The GMV of each voxel was obtained by multiplying the GM concentration map by the non-linear determinants derived during spatial normalization. Finally, the GMV images were smoothed with a full-width at half-maximum (FWHM) kernel of 8 mm to compensate for residual between-subjects anatomical differences. The resulting images had a normalized voxel size of 1.5 × 1.5 × 1.5 mm. After spatial pre-processing, normalized, modulated, and smoothed GMV images were used for the following statistical analysis.

### Statistical analyses

**Fig. 1:**
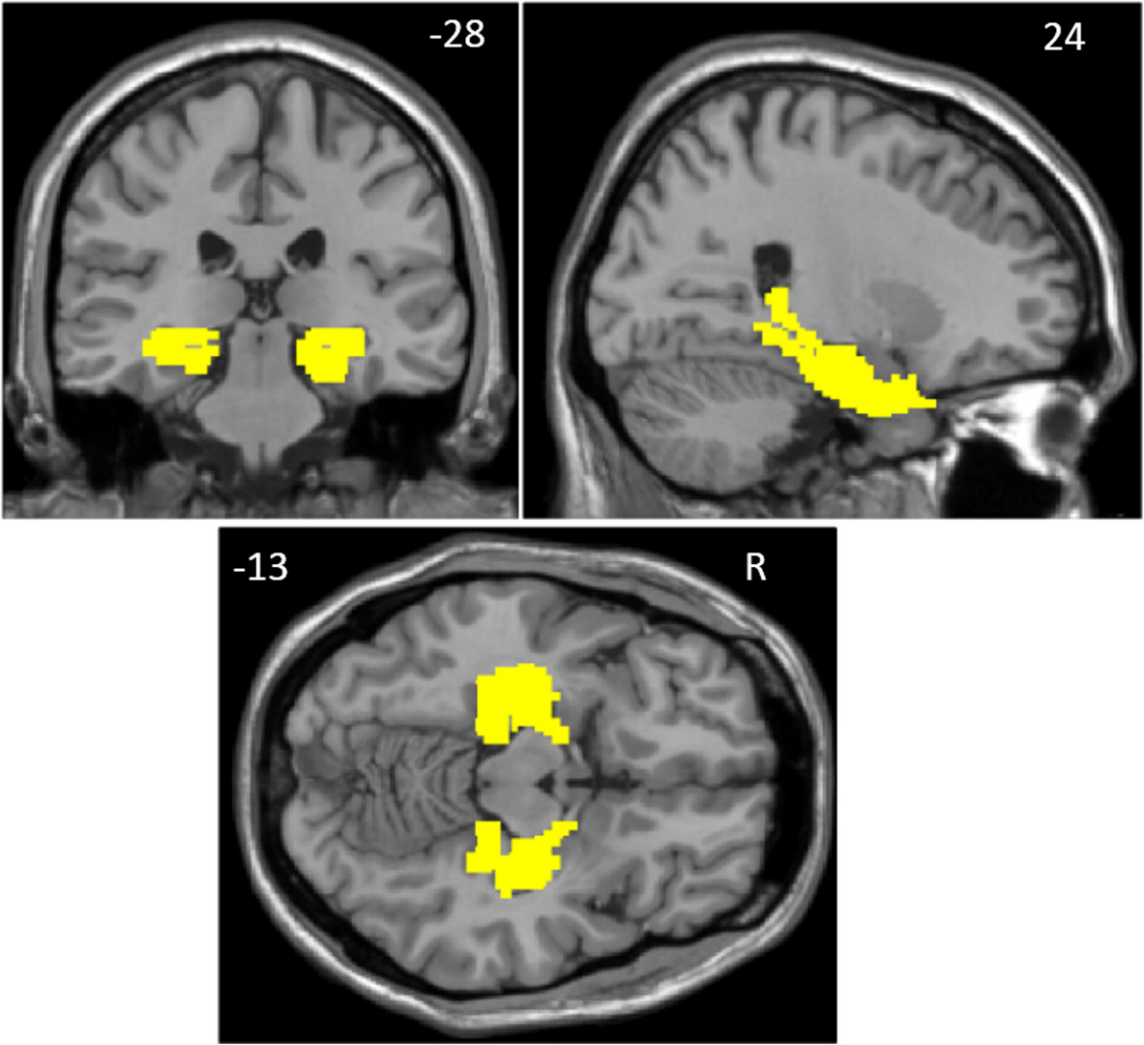
Bilateral hippocampal/parahippocampal regions of interest (yellow) defined through the automated anatomical labeling atlas. Images are in radiologic format with the left hemisphere on the right side of the image

Preceding statistical analysis, a priori region of interest (ROI) including the bilateral hippocampi and parahippocampi was defined according to the automated anatomical labeling atlas (AAL) from Wake Forest University (WFU) PickAtlas toolbox (http://www.Nitrc.org/projects/wfu_pickatlas) and used for the following two-sample *t*-test. Figure 1 shows the slice view of the ROI applied to each of the 36 subjects.

A two-sample *t*-test within the predefined ROI was performed between the two groups with the aim to investigate whether there were any significant regional GMV differences in hippocampus/parahippocampus between StD and HC subjects. To control for possible confounding variables, age, gender, education level, MMSE, and total intracranial volume (TIV) were entered as covariates. A cluster threshold at *α* < 0.05 (AlphaSim-corrected) for multiple comparisons was considered significant. This correction entailed a primary threshold of *p* < 0.01, with an extent threshold of 121 voxels (FWHM = 12.3 mm; cluster connection radius *r* = 2.5 mm; with the ROI mask and a resolution of 1.5-mm^3^).

In order to investigate whether the altered GMV of the ROI was correlated with the presence of a subthreshold depression, a correlation analysis between the GMV of the ROI and participants’ self-reported depressive symptoms was also performed. CES-D score was used as a behavioral variable, with the above-mentioned variables as confounding covariates. In addition, the correlation coefficients of the relationship between the participants’ self-reported depressive symptoms and GMV extracted from the ROI were also calculated.

## Results

Table 1 shows the demographic characteristics and neuropsychological scores of each group. There were no significant differences in age (*p* = 0.6), gender (*p* = 0.8), and years of education (*p* = 0.96) between the HC and StD group. No significant difference on MMSE score was found between the groups (*p* = 0.82). Therefore, the two groups were matched on the basic cognitive level. Merely the variable of interest, the CES-D score, was significantly higher in the StD group than in the control group (*p* < 0.01). This study has adhered to the STROBE (Strengthening the Reporting of Observational Studies in Epidemiology) reporting guidelines of cross-sectional data Additional file 1.

**Table 1:** Demographic and clinical characteristics of elderly subjects with subthreshold depression and healthy age-matched controls

Characteristics	HC	StD	*p* value
Gender(M/F)	17(7/10)	19(7/12)	0.8^*^
Age, years	66.5 ± 4.0	66.5 ± 5.7	0.6^**^
Education, years	13.8 ± 2.6	13.2 ± 2.7	0.96^**^
MMSE	28.9 ± 1.5	28.3 ± 1.6	0.82^**^
CES-D	1.1 ± 1.6	16.1 ± 5.1	<0.01^**^

^*^ *p* Value for gender distribution was obtained by χ^2^ test

^**^ *p* Values were obtained by *t*-test

### ROI GMV alterations in StD

The two-sample *t*-test showed that StD participants had significantly reduced volumes of gray matter in the right parahippocampus in comparison to HC participants (peak MNI coordinate: 30, -23, -27, *t* = 3.96, *p* < 0.01; number of voxels: 201, Fig. 2). The Cohen’s d for effect size is 0.88.

**Fig. 2:**
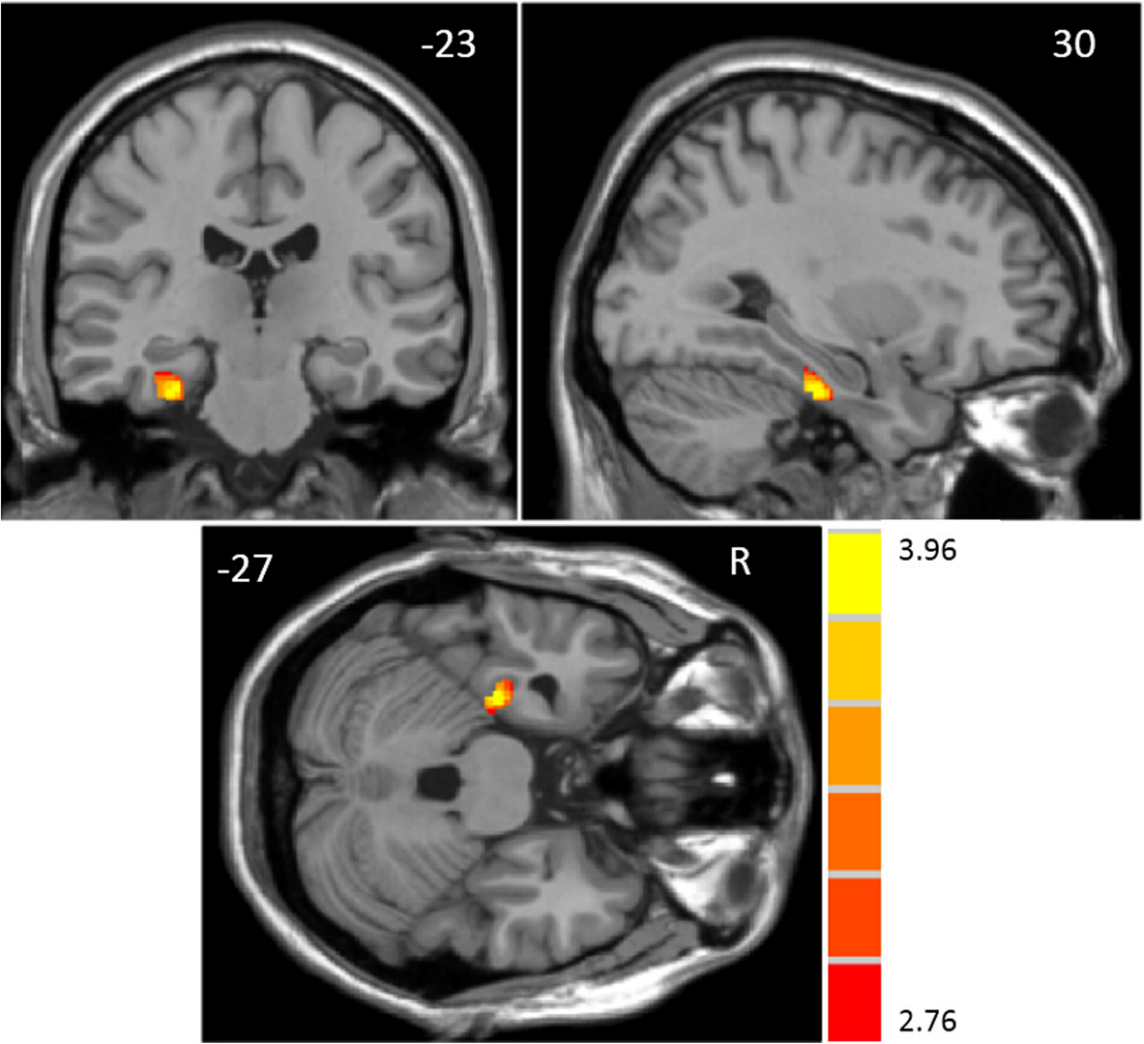
Gray matter volume reductions in the right parahippocampus (peak MNI coordinate: 30, -23, -27, *t* = 3.96, *p* < 0.05) among older adults with subthreshold depression compared to healthy controls. The *t* - values are shown on the color bar

### Correlation between ROI GMV and self-reported depressive symptoms

**Fig. 3:**
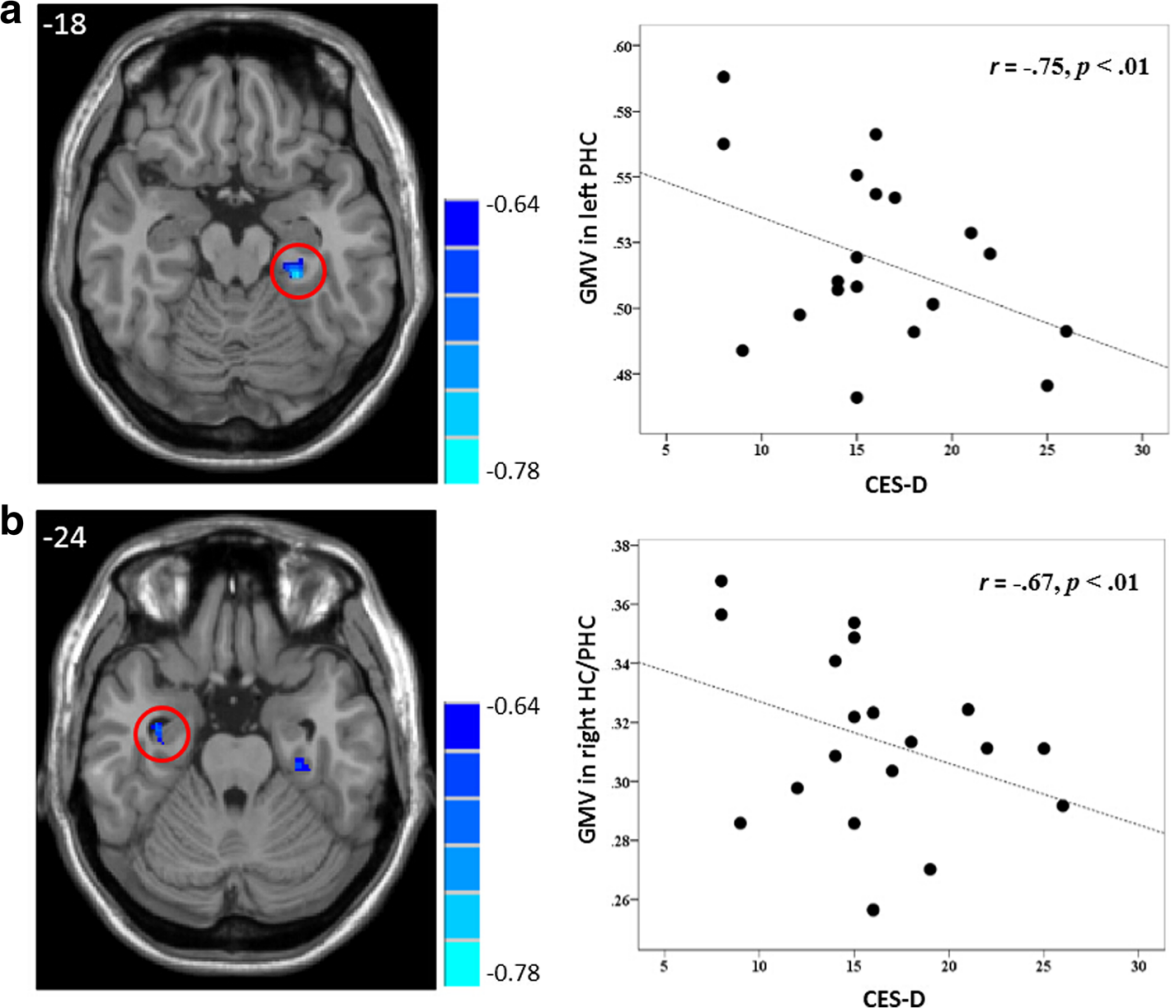
The brain region map and the scatter plots of the correlation between the GMV in the left parahippocampus (**a**) and the right hippocampus/parahippocampus (**b**) and the Center for Epidemiologic Studies Depression scale scores in individuals with subthreshold depression. The *r* - values are shown on the color bar. Each circle dot represents the data of one participant. The GMV of the hippocampus/parahippocampus was obtained by defining a sphere centered at the voxel showing the highest statistical differences, with a radius of 6 mm

The correlational analysis showed that the CES-D score in the StD group was inversely correlated with the volumes of the left parahippocampus (peak MNI coordinate: -27, -33, -18, *t* = -0.78, *p* < 0.01; number of voxels: 147; *r* = -0.75, *p* < 0.01, AlphaSim corrected, Fig. 3a). With a more liberal primary threshold of *p* < 0.01 and a cluster size of at least 30 voxels, the results showed that the CES-D score in the StD group was inversely correlated with both the volumes of the right hippocampus/parahippocampus (peak MNI coordinate: 36, -8, -24, *t* = -0.71, *p* < 0.01; number of voxels: 35; *r* = -0.67, *p* < 0.01, Fig. 3b) and the left parahippocampus (peak MNI coordinate: -27, -33, -18, *t* = -0.78, *p* < 0.01; number of voxels: 147; *r* = -0.75, *p* < 0.01, Fig. 3a). The scatter plots of correlation between the hippocampus/parahippocampus GMV and participants’ self-reported depressive symptoms were reported in the same figure (Fig. 3). As can be seen there is a significant negative correlation between the left parahippocampus and right hippocampus/parahippocampus GMV and participants’ self-reported depressive symptoms.

## Discussion

By using voxel-based morphometry analysis, the current study investigated structural alterations of the hippocampus/parahippocampus and potential correlations between its volume and participants’ self-reported depressive symptoms in the elderly suffering from subthreshold depression. The results showed that elderly people with StD had substantially reduced volumes of the right parahippocampus compared with healthy age-matched controls. Moreover, the volumes of the hippocampus/parahippocampus were significantly correlated with participants’ self-reported depressive symptoms in StD. These results demonstrate that structural alterations of the hippocampus/parahippocampus may occur at an early stage of depression.

Consistent with our hypothesis, after controlling age, education level, cognitive functioning (assessed by means of the MMSE), and total intracranial volume, the parahippocampal volume reductions were observed only in elderly subjects with StD. These results are in line with those of elderly patients suffering from major depressive disorders [12–16]. Importantly, findings of the present study highlighted a relation between structural alterations of the hippocampus/parahippocampus and a precursor of major depression, the subthreshold depression, in older adults. Interestingly, some previous volumetric investigations that only recruited male elderly with subclinical depression [21] and also older adults with depressive symptoms [22] failed to find such variations. It is important to note that the results of these previous studies may have been potentially confounded by factors such as differences in demographic and clinical characteristics of the samples, the kind of depression measurements, and also the usage of different analytic strategies. In [22], CES-D scores (*Mean* = 6.37, *SD* = 2.04) were considered as a measure of the chronic or persistent depressive symptoms. Comparing their scores with those obtained in the here presented study, their participants were less depressed than ours (CES-D *Mean* = 16.05, *SD* = 5.06). Furthermore, participants in [22] entered the study at an age between 56 and 85 years at baseline and considering that this longitudinal study lasted 9 years it should be kept in mind that their participants were 64–97 years old at the final evaluation. In contrast, our participants had an age range between 58 and 75 years at the time of investigation which represents a definitely narrower age window than that of [22]. Such a narrow age range of our participants minimizes the aging effect of gray matter loss in cerebral cortices regarding StD-dependent changes. With respect to the usage of different measurement tools to assess depression, [21] defined participants with scores on the Geriatric Depression Scale (GDS) of 22 and higher as subthreshold depression. In contrast, we used CES-D to define the subthreshold depression in the elderly. Such differences in measuring depression may also contribute to the inconsistent findings between studies. Moreover, [21] conducted a whole-brain analysis, we, however, carried out a region-of-interest analysis. The usage of different analytic strategies may also lead to different results. Such inconsistencies between our study and previous studies also emphasized the urgent need for more evidence on brain structural variance associated with subthreshold depression. Our study provides one relevant jigsaw piece, future studies should however address other related details in order to gain a more comprehensive picture of the neuronal underpinnings of depression.

Altered hippocampal/parahippocampal connectivity with prefrontal and cuneus cortices in one of our previous studies has also been reported [19] showing that the elderly with StD exhibit decreased hippocampal/parahippocampal connectivity to the superior and medial frontal gyri compared to unaffected controls. Similarly, decreased hippocampal-DLPFC functional connectivity was also reported [17]. These regions represent crucial components of the cortical-limbic-subcortical circuit [7]. Such findings demonstrated that the top-down control of the prefrontal cortex over limbic regions in older adults suffering from MDD or StD is attenuated which as a consequence may cause a depression according to the cortical-limbic-subcortical dysregulation model of depression [7, 9]. Such mechanisms are most likely considering that the cortical-limbic-subcortical circuit receives major afferents from the hippocampus/parahippocampus [32]. Disruption within the gray matter of anatomic connections between parts of the frontal-subcortical limbic system may lead to functional, and ultimately structural, impairment of the hippocampus/parahippocampus [33, 34] which may result in depression [35]. Previous studies also showed impaired cognitive control of emotional stimuli in individuals with depressive symptoms [36] and structural and functional changes in the frontal and temporal regions in patients affected by subthreshold depression [21, 22, 37].

The hippocampus is involved in the regulation of the hypothalamic pituitary adrenal (HPA)-axis which is responsible for the production of stress-related glucocorticoids such as cortisol [38]. It has also been suggested that parahippocampus and hippocampal connections with other subcortical structures may play a key role in the regulation of stress [39]. Indeed, depressed individuals have been found to have high levels of stress [40] which is reflected biologically in elevated rates of hypercortisolemia and disturbed HPA-axis functioning. Furthermore, it has been reported that neurons of the hippocampus are highly sensitive to the deleterious effect of increased glucocorticoid levels which are, in turn, associated with repeated stressful episodes [41]. Studies also revealed that reduced hippocampal volume is associated with increased cortical secretion in response to stressors [42], thus implying an association between hippocampal volume reduction and elevated cortisol levels. Given the association between depression and glucocorticoid production, it is not surprising that volume reduction of the hippocampus is observed in populations with MDD. One of the possible reasons for the similar finding of hippocampal/parahippocampal volume reduction in the present study may also be related to the exposure to repeated episodes of hypercortisolemia and disturbed HPA-axis functioning [43]. Sustained hypercortisolemia causes down-regulation of glucocorticoid receptors and ultimately prevents hippocampal neurogenesis [44].

## Conclusions

In conclusion, the present study demonstrates that reduced volume in the hippocampus/parahippocampus is significantly associated with StD suggesting that early structural changes in the hippocampus/parahippocampus can constitute a risk indicator of MDD. However, the present study is a cross-sectional one. The findings suggest that reduced hippocampal/parahippocampal volume might be considered a precursor of a depression onset. Following this reasoning, such early information may impact the diagnosis of depression at the preclinical stage. It is also possible that reduced hippocampal/parahippocampal volume may not be directly related to the development of a later depression but rather implies a preexisting vulnerability factor for depression. Because the present study was cross-sectional such an explanation cannot be ruled out. Longitudinal studies following the progression of depression (from normal states to subthreshold depression towards a clinical depression) might help to solve these problems. Moreover, our inferences of the relationship between structural alternations in the hippocampus/parahippocampus and StD are drawn from a small sample which may have increased the likelihood of a type I error (false positive). Therefore, these findings should be considered preliminary so far necessitating replication from larger samples.

## Abbreviations

CES-D, Center for Epidemiologic Studies Depression scale; CSF, cerebrospinal fluid; DLPFC, dorsolateral prefrontal cortex; FWHM, full-width at half-maximum; GMV, gray matter volume; HC, healthy age-matched control; HC, hippocampus; HPA, hypothalamic pituitary adrenal; MDD, major depressive disorders; MMSE, Mini Mental State Examination; MNI, Montreal Neurological Institute; MRI, Magnetic Resonance Imaging; PHC, parahippocampus; ROI, region of interest; StD, subthreshold depression; TIV, total intracranial volume; VBM, voxel-based morphometry

## Additional file

Additional file 1: STROBE Statement—Checklist of items that should be included for cross-sectional studies (XLSX 14 kb)
